# One Health and Australian Aboriginal and Torres Strait Islander Communities: A One Health Pilot Study

**DOI:** 10.3390/ijerph20146416

**Published:** 2023-07-20

**Authors:** Tamara Riley, Bonny Cumming, Joanne Thandrayen, Anna Meredith, Neil E. Anderson, Raymond Lovett

**Affiliations:** 1 National Centre for Epidemiology and Population Health, The Australian National University, Canberra, ACT 2601, Australia; 2 Animal Management in Rural and Remote Indigenous Communities (AMRRIC), Darwin, NT 0801, Australia; 3 The Royal (Dick) School of Veterinary Studies and the Roslin Institute, University of Edinburgh, Roslin EH25 9RG, UK

**Keywords:** One Health, Aboriginal and Torres Strait Islander, human health, animal health, environmental health, zoonoses

## Abstract

Many Aboriginal and Torres Strait Islander communities face barriers in accessing animal healthcare and are exposed to disproportionate environmental health exposures leading to increased risk of disease. A One Health approach has been promoted to address public health risks and improve human, animal, and environmental health outcomes in communities. We undertook a pilot One Health study in Aboriginal and Torres Strait Islander communities in Queensland collecting animal, human, and environmental health data from 82 households. We performed a descriptive analysis and assessed the association between human and environmental health exposures and animal health outcomes. Most households were not crowded (82.9%) but did report a high level of environmental health concerns (86.6%). The majority of households owned cats and dogs (81.7%), with most animals assessed as healthy. There was no association between human and environmental health exposures and animal health outcomes. As most households experienced concerns regarding housing conditions, environmental health programs should prioritise improving household factors. There was also strong support for animal healthcare (including access to medicines and veterinarians, education programs and population management), indicating that a One Health approach is desired by communities.

## 1. Introduction

Internationally, multiple disease outbreaks of zoonotic origin have shown the importance of understanding the human–animal–environmental health relationship (One Health). One Health is “an integrated, unifying approach that aims to sustainably balance and optimise the health of people, animals, and ecosystems. It recognises the health of humans, domestic and wild animals, plants, and the wider environment are closely linked and interdependent” [1] (p.2). This concept is promoted by the Quadripartite Alliance as an effective and sustainable approach to addressing public health risks globally, including the control of emerging zoonotic diseases [2,3,4]. However, the implementation of the concept is not widely reported, and increased collaboration and communication between health sectors; the integration of health systems across sectors; and improved knowledge translation between researchers, policymakers, and communities are needed [5,6]. While the main focus of the concept is infectious disease, including zoonotic diseases (diseases that pass between animals and people), other ailments, such as chronic disease, mental health, injury, occupational health, and non-communicable diseases, can also benefit from a One Health approach [5,7]. One Health also recognises relationships regarding emotional connection and companionship between people and animals as well as the environmental impacts of domestic animals (including on wildlife populations and habitats) [8,9].

One Health is highly relevant to Aboriginal and Torres Strait Islander health in Australia and is aligned with community and cultural values that recognise the integral relationships between the health of people, animals, and the environment [10]. However, many Aboriginal and Torres Strait Islander communities face a high risk of disease related to the environment and animals, and a One Health approach is likely to assist in combating this [11,12]. When considering One Health at a community level, people’s physical, social, and spiritual connections to animals and the environment are well recognised [13,14,15]. In many communities, people and animals live closely together, yet access to effective animal healthcare and associated environmental health practices is extremely limited. This can lead to many public health concerns, including large and unmanaged animal populations [16], animal health and welfare concerns [8], environmental damage and degradation [9], and impacts on wildlife populations [17]. 

Aboriginal and Torres Strait Islander communities are disproportionately exposed to environmental health risks, such as poor infrastructure, water and air quality, and contact with wild and domestic animals [18]. Housing conditions are of particular concern for many communities, with inadequate housing and overcrowding leading to an increased risk of infectious diseases and poor health outcomes [19,20,21]. Communicable diseases, including zoonotic diseases, are also prevalent in the Aboriginal and Torres Strait Islander population, some of which are described as ‘diseases of poverty’ globally, for example, strongyloidiasis [22,23,24,25]. These inequalities are likely to increase due to climate-related health risks, including weather extremes and increased contact with vectors [26,27]. This is particularly the case in tropical areas of Australia that face an increased risk of introduced zoonoses, such as rabies, from neighbouring countries to the north of Australia [13,28]. Injuries related to the environment and animals are also a concern for Aboriginal and Torres Strait Islander communities, with large domestic animal populations in remote areas and a high incidence of related injury presentation, such as dog bites, in health clinics [16,29,30].

While highly relevant, the One Health paradigm and its contribution to Aboriginal and Torres Strait Islander health are neither well understood nor commonly practised in Australia; minimal evidence is available, limiting our ability to address the needs of communities effectively [10]. The current siloed health systems approach also limits our ability to address these issues with One Health considerations [5]. To assist with this, One Health databases that can manage data from the animal, human, and environmental health sectors together would be beneficial; however, this has not yet been investigated. There is also a need to increase the scope of One Health research outside of animal and human health, as environmental health is frequently underrepresented [10]. This would help to address the factors that lead to an increased risk of disease and injury related to the environment and animals [25]. This is relevant to the United Nations Sustainable Development Goals that aim to reduce environmental health risks, improve access to healthcare and health outcomes, and have been linked to animal welfare, including dog population management [31,32,33].

We undertook a pilot One Health study across three discrete Aboriginal and Torres Strait Islander communities that included components from animal, human, and environmental health [34]. We designed and implemented a One Health data collection and analysis framework to improve understanding of risks across the three domains of One Health. To our knowledge, this is the first study to design and implement a One Health data framework with Aboriginal and Torres Strait Islander communities in Australia.

## 2. Materials and Methods

### 2.1. Ethics

The study was conducted in accordance with the Australian Institute of Aboriginal and Torres Strait Islander Studies (AIATSIS) Research Ethics Committee (EO243-20210406).

### 2.2. Study Design

This study was undertaken by an Aboriginal-led multidisciplinary research team using Indigenous research methodology and a strength-based approach to foster Aboriginal and Torres Strait Islander leadership, prioritise Indigenous voices, and strengthen the reporting of health research [35,36,37]. We undertook this study in partnership with Indigenous Local Government Authorities and Animal Management in Rural and Remote Indigenous Communities (AMRRIC), a national not-for-profit organisation that works with communities to support and deliver animal healthcare and education programs [38]. We designed an ecological study using a One Health data framework to collect and analyse information on animal, human, and environmental health factors at a household level [34]. An ecological study design was an appropriate approach, as it allowed the consideration of multiple One Health factors within the household environment rather than focusing on individual factors [39,40]. We considered the One Health sectors as follows: ‘animal’ referred to domestic animals; ‘human’ referred to people; and ‘environment’ referred to ecosystems, including the physical environment, plants, wildlife, and invertebrates. 

The data collection tool (household survey) was designed through an iterative process by first using previous research and evidence to draft the survey, highlighting key areas of interest and workshopping ideas with community organisations [30]. We limited the survey to questions related to health risks associated with barriers to accessing animal healthcare and associated environmental health exposures (such as the risk of zoonotic disease). We further workshopped the survey questions with the research team and AMRRIC and sought feedback from community Animal Health Workers. Following feedback during data collection, we also adjusted the survey items by changing the language used and the delivery of the survey to make the survey appropriate for the context. After each data collection, we had a feedback session with the research team to assess preliminary results, discuss how the questions were received, and update the survey items as needed. 

### 2.3. Data Collection

Data were collected from March to July 2022 in three regional and remote communities in Queensland, Australia. The Aboriginal and Torres Strait Islander population of Queensland represents 4.6% of the state’s entire population and has an average of 3.2 people per household [41]. Comparatively, the three communities had Aboriginal and Torres Strait Islander populations of up to 98%, with populations of 1000–2500 people per community and approximately four people per household [41]. This study represented 7–10% of the households in each community.

The household survey included initial identifying questions (address and Aboriginal and Torres Strait Islander status), followed by questions from within the three One Health sectors and questions involving the interaction between two sectors (human and animal health; human and environmental health) (Supplementary Table S1). The animal health assessments collected information on the health of the cat and dog population in each household. These included demographic factors and health indicators, such as body condition (scored from 1 to 9), hair and skin condition (scored from 1 to 6), and ticks and fleas (scored from 1 to 4). We also delivered community-wide preventive animal health programs and animal population census simultaneously. While all communities faced limited access to animal health and management, each community had a variable history and frequency of services.

To collect data, we visited households and undertook animal health assessments of the cats and dogs, collecting this information digitally using the AMRRIC mobile application, a custom-designed companion animal population data collection tool utilised by AMRRIC for animal health service delivery [42]. We simultaneously delivered the household surveys by asking questions about human and environmental health exposures (including animal husbandry) related to animal health outcomes. The household surveys were undertaken by one data collector (TR), and the animal health assessments were undertaken by multiple data collectors (including TR, AMRRIC, and Animal Health Workers). The surveys were delivered using the Redcap Electronic Data Capture tool hosted at the Australian National University. Redcap is a secure, web-based platform designed to support data capture for research studies, providing an interface for validated data capture; audit trails for tracking data manipulation and export procedures; automated procedures for export to statistical packages; and procedures for data integration with external sources [43,44].

### 2.4. Data Analysis

**Figure 1:**
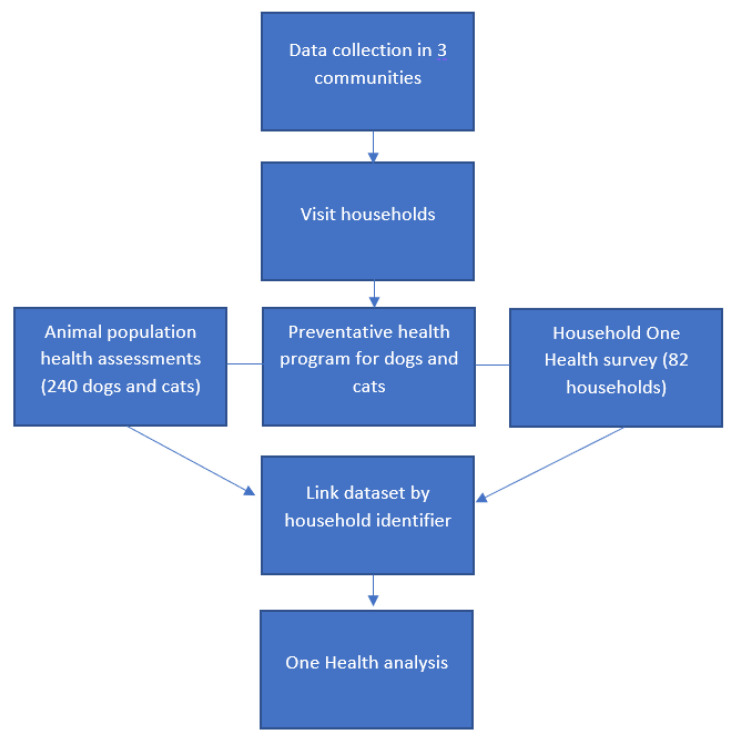
Fieldwork and data processing flow chart.

Data were cleaned and analysed using Excel and Stata 17, ensuring the anonymity of households. We linked the data by the household identifier to create a One Health household ecological dataset that included animal, human, and environmental health factors (Figure 1). We summarised the individual animal health data and averaged the body condition scores, hair scores, and tick and flea scores at the household level. We initially undertook a descriptive analysis reporting frequencies, percentages, and free responses where applicable. We then grouped the human and environmental health exposures and animal health outcome variables into binary categorical groups (Supplementary Table S2). Exposures included human health (household crowding, perceived crowding, and concerns for animal health) and environmental health (household environmental concerns, animal crowding, and animal breeding). The animal health outcomes included healthy or unhealthy animals, as assessed by body condition score, hair score, and tick and flea scores.

To assess households for human health exposures, crowding was calculated using the Canadian National Occupancy Standard Criteria, which state that the acceptable requirements are ‘no more than 2 people per bedroom per household’, on average [45]. We calculated the average number of people per bedroom per household to assess crowding and grouped the data into ‘>2 people per bedroom per household’ (crowded) and ‘≤2 people per bedroom per household’ (not crowded). We also grouped the perceived crowding variable into ‘crowded’ (yes, a lot; yes, a fair bit; yes, a little bit) and ‘not crowded’ (does not feel crowded). For concerns about animal health, we grouped the variable into ‘current concerns for animal health’ (yes, a lot; yes, a fair bit; yes, a little bit) and ‘no concerns’ (does not feel concerned). 

To assess households for environmental health exposures, we grouped households into ‘concerns for household environment’ (at least one household environmental concern) and households with ‘no concerns’. We assessed the households for animal crowding by calculating the total number of cats and dogs in each household and grouping households into those with 1 to 4 animals (‘no animal crowding’) and those with over 4 animals (‘animal crowding’). We also assessed households for animal breeding by grouping households into those that had litters of puppies and kittens present (‘animal breeding’) and those that did not (‘no breeding’). 

For the animal health outcomes, we grouped the body condition score into ‘healthy BCS’ (BCS of 4 to 6, considered ideal) and ‘unhealthy BCS’ (BCS of 1 to 3, considered thin, or a BCS of 7 to 9, considered overweight). We grouped hair scores into ‘healthy hair score’ (hair score of 1, indicating no hair loss) and ‘unhealthy hair score’ (hair score of 2 to 6, indicating 20% hair loss to 100% hair loss). Similarly, we combined flea and tick scores and grouped the variable into ‘healthy tick and flea score’ (tick and flea score of 1, indicating no ticks and fleas) and ‘unhealthy tick and flea score’ (tick and flea score of 2 to 4, indicating mild to severe ticks and fleas). For all variables, missing data were not included in the analysis. 

As variables were binary categorical variables with small cell sizes (Supplementary Table S3); we used the Fisher’s exact test to assess the association between the exposures (human health and environmental health) and the outcomes (animal health) with a *p*-value < 0.05 considered significant [46].

## 3. Results

We undertook data collection across 82 households in three communities. Almost all households (98.8%) were exclusively Aboriginal and Torres Strait Islander households (Supplementary Table S4). 

### 3.1. One Health Concept Factors

Almost half (41.5%) of the households answered ‘yes’ to animal health affecting householders health, and 11% of the responses were missing (Supplementary Table S4), with the most common relationships recognised as ‘affects people’s wellbeing (sad when animals are sick and injured)’ (15.8%), ‘sick animals make people sick ‘(14.6%), and ‘itchy animals make people itchy’ (11.0%). When asked about animal and householders health relationships, community members mentioned the following well-being interactions:

- Helps when you are sick in a good way;
- Kids like the dogs and feed and water them;
- We do worry about them becoming sick and old;
- My daughter would be sad without her dogs;
- Have arguments with other people about my dogs when they bark;

In addition, they mentioned the following health interactions:

- Sick dogs make kids sick—they play with them and kiss them;
- Hygiene of dogs, rolling around in whatever and coming inside;
- When they poo with worms in them;
- Skin sores, too many animals;
- Scabies, ticks and fleas, and whatever else they have.

Only a quarter (23.2%) of households recognised relationships between environmental health and householders health, and 29.3% did not; however, 43.9% of households had missing data for this question (Supplementary Table S4). Of those who did recognise a relationship, the following were mentioned:

- Sick environments make people sick;
- If the environment is not good, dogs are not going to be good because they live off the environment;
- Waste thrown everywhere, going into waterways and landfill;
- So many cats affecting and hunting wildlife, do not see frogs around anymore;
- Long grass, insects after rain;
- Heat, pollution.

### 3.2. Human Health Factors

**Figure 2:**
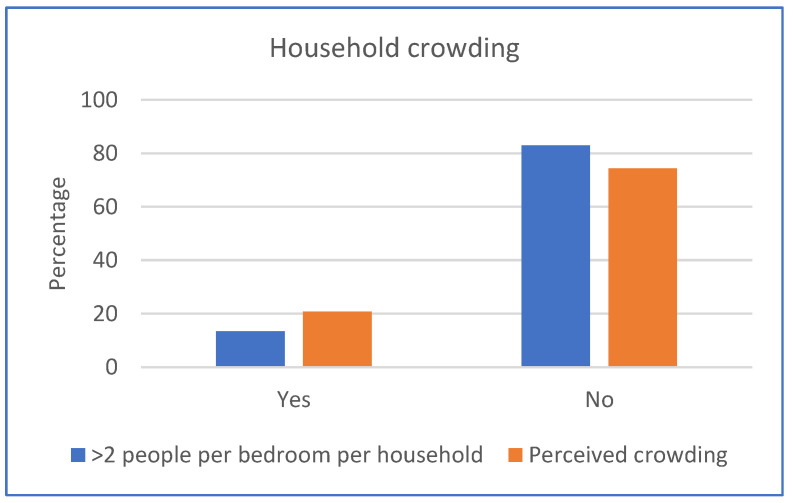
Calculated and perceived household crowding.

Households had an average of 4.9 inhabitants and a median of four inhabitants and four bedrooms. Around a third of households had three to four people per household (31.7%) and four bedrooms (39.0%) (Supplementary Table S4). Across all three sites, there was an average of 1.5 people per bedroom, with most households classified as not crowded (82.9% of households had two or fewer people per bedroom) and 13.4% of households having more than two people per bedroom, with 3.7% of households missing data. The majority of people also felt that their households were not crowded (74.4%), with 20.7% answering yes to the house feeling crowded (Figure 2).

When respondents were asked if they had current concerns about the health of their animals, over half of them said ‘yes’ (52.4%), and 45.1% did not have concerns (Supplementary Table S4). For those who said yes, households were concerned about the risk of disease (30.5%) and injury (11.0%). People mentioned the following about current concerns for animal health:

- Worried about them becoming sick and becoming older;
- Injured and cannot help them;
- Worms, ticks, fleas, having too many puppies;
- Skinny and have to wait for payday to buy feed.

### 3.3. Environmental Health Factors

**Figure 3:**
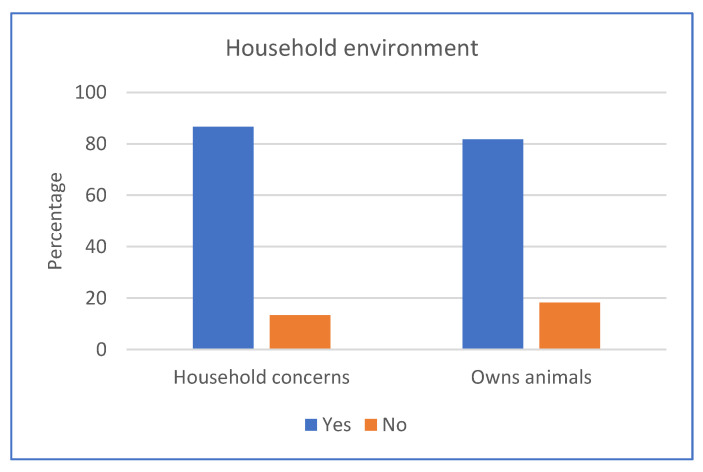
Household environmental factors.

Most households had concerns with their household environment (86.6%) (Figure 3), including fencing to contain animals in yards (57.3%) and pests, such as rodents, insects, and parasites (63.4%) (Supplementary Table S4). Community members mentioned:

- Need high fences to keep animals in;
- Problems keeping the other dogs out, they jump over the fence and steal my dog’s food;
- Rats, snakes, cockroaches, and ants;
- Ticks climbing on the walls.

Wild animals (domestic species) were also a concern for some households (29.3%), with wild bulls, wild horses, and wild dogs mentioned.

Most households (81.7%) owned cats and dogs, with a total of 240 animals reported (Figure 3, Supplementary Table S4). Most households owned one to four animals (58.5%), with 17.1% owning five to eight and 6.1% owning nine or more animals. Approximately 1 in 10 households (11.9%) had litters of puppies or kittens, signalling animal breeding. 

### 3.4. Animal Health Factors

**Figure 4:**
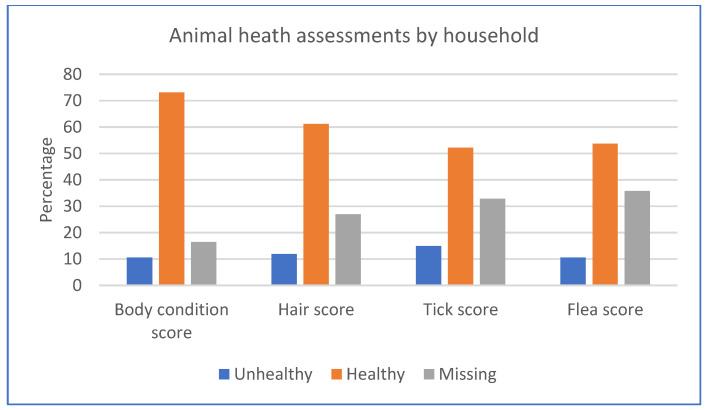
Animal health assessments.

Out of the 81.7% of households that owned cats and dogs, 73.1% of households had animals with ideal body condition scores (BCS of 4–6), 61.2% had animals with healthy hair scores (hair score of 1), and 52.2% and 53.7%, respectively, had animals with no ticks and fleas (tick and flea score of 1) (Supplementary Table S4). For each measure, less than 15% of households were assessed as having animals with unhealthy outcomes; however, these measures had many missing data (Figure 4).

Almost all households reported needing more animal healthcare within their communities. Household priorities regarding animal healthcare included having animal medicines available (51.2%), having more vets visiting the community (40.2%), and having regular and ongoing animal health programs (31.7%) (Supplementary Table S4). When discussing animal healthcare, people mentioned:

- Not having anything here like in other places to help animals;
- Desexing dogs and horses, ongoing education for kids on how to treat animals;
- Dog wash visits;
- Anything, just more healthcare;
- Shops that sell more stuff for dogs;
- Help with injured animals, do not have resources;
- Would be good to have more vet visits.

### 3.5. Relationships between Human and Environmental Health Exposures and Animal Health Outcomes

Regarding human health exposures, it was most common for animals with a healthy BCS to live in non-crowded households (89.4%), households perceived as non-crowded (74.5%), and households with current concerns for animal health (52.1%). Regarding environmental health exposures, it was most common for animals with a healthy BCS to live in households with environmental concerns (87.8%), no animal crowding (69.4%), and no animal breeding (83.7%). However, the associations between human health and environmental health exposures and animals with a healthy BCS were not significant (Table 1).

Regarding human health exposures, it was most common for animals with healthy hair scores to live in non-crowded households (92.3%), households perceived as non-crowded (76.9%), and households with current concerns for animal health (55.0%). Regarding environmental health exposures, it was most common for animals with healthy hair scores to live in households with environmental concerns (87.8%), no animal crowding (68.3%), and no animal breeding (82.9%). However, the associations between human health and environmental health exposures and animals with healthy hair scores were not significant (Table 2).

Regarding human health exposures, it was most common for animals with healthy tick and flea scores to live in non-crowded households (90.6%), households perceived as non-crowded (75.0%), and households with no current concerns for animal health (54.5%). Regarding environmental health exposures, it was most common for animals with healthy tick and flea scores to live in households with environmental concerns (84.9%), no animal crowding (63.6%), and no animal breeding (81.8%). However, the associations between human health and environmental health exposures and animals with healthy tick and flea scores were not significant (Table 3).

## 4. Discussion

This is the first study to implement a One Health data collection and analysis framework in Aboriginal and Torres Strait Islander communities. As the One Health concept was central to this work, we investigated how the concept resonates within communities by asking about the relationship between household, animal, and environmental health. While less than half of households stated that they recognised the interaction between sectors, this variable had many missing data. This may be related to the language and delivery of the questions, as these One Health relationships can be construed in many ways, and more consideration is needed to further investigate this aspect. Furthermore, survey items related to the interactions between animal and environmental health and all three One Health sectors would be helpful in future iterations.

Most households were classified as not crowded; however. more households were perceived as such, with a fifth of households answering that their household felt crowded. While the Canadian National Occupancy Standard is frequently used to calculate crowding, it can be argued that this is not an appropriate measurement for Aboriginal and Torres Strait Islander communities; a new method that considers social and cultural factors is warranted [45]. To combat this, we also asked participants about perceived crowding to avoid relying solely on the crowding calculation. Evidence has shown that crowded households are a common concern for Aboriginal and Torres Strait Islander communities, contributing to poor health outcomes; therefore, the collection of this data is useful for assessing public health risks within communities [20,47].

We asked about people’s concerns for animal health due to the close connection many families have with their animals and the associated well-being impacts [8]. Over half of households had current concerns about the health of their animals, noting that most animals were healthy; however, risks of disease and injury in animals were commonly reported. Limited animal healthcare services and animal products, including food, medicines, and animal husbandry items, were noted, limiting owners’ abilities to keep their animals healthy and highlighting a need for these services within communities. This could be related to socioeconomic barriers, with some owners noting they could not access animal health services, medicines, and products when they were available due to cost constraints. 

The environmental health questions concentrated on household environmental health exposures and included common issues discussed within the evidence base, such as housing conditions, hygiene, and sanitation factors [20,48]. Most households had concerns with their environment, with poor fencing reported as a factor limiting the ability of owners to control their animals’ environment and reduce the risk of disease and injury, as animals could freely roam. Pests, including ticks, mites, fleas, and rodents, which are reservoirs for disease, were commonly reported, with preventive controls not readily available. These findings are in line with evidence that recommends improved housing conditions for Aboriginal and Torres Strait Islander households to prevent communicable diseases and subsequent poor health outcomes [19,47]. With most households reporting environmental concerns and a fifth of households feeling that their households were crowded, addressing housing concerns should be prioritised.

Additionally, exposure to and interactions with wild animals is of interest, as wildlife can be reservoirs for zoonotic diseases [2]. Domestic wild animals were mentioned by a quarter of households as a concern for the household environment. Comparatively, the effect of the domestic animal population on wildlife populations and habitats needs further investigation, as large populations of free-roaming domestic animals are likely to negatively impact native wildlife, for example, through predation [17]. Hunting can also be a usual practice in communities, bringing people and domestic animals close to wild animal populations and leading to public health risks [49]. Due to the iterative design of the survey, we asked about hunting in only two out of three communities and, therefore, did not report these results. We also asked about where animals lived within the household, as this relates to peoples’ relationships with their animals and can be an environmental health exposure; however, as we only asked this in two out of three communities, we also did not report these results.

The majority of households did not have animal crowding. While we used four animals as the cut-off to assess crowding, the policies around animal ownership in Australia vary by state and community, with no consistent guidelines on how many animals lead to crowding. As Aboriginal and Torres Strait Islander communities have high percentages of pet ownership and large animal populations [16,50], this needs further investigation to recognise whether the number of animals per household affects health and wellbeing. Most households had dogs and cats assessed as healthy, and almost all households reported needing more animal healthcare services; the most common priorities were regular and ongoing access to animal medicines and veterinary services. However, these services need to be accessible to low-income families and readily available. Queensland state legislation also requires desexing of registered dogs and cats, further supporting the need for resourcing of animal management programs [51].

Seeking community members’ input on priorities to inform future initiatives is in line with Indigenous research methodologies and aligns with the United Nations Declaration on the Rights of Indigenous Peoples, which aims for self-governance and Indigenous leadership within local community contexts [37,52]. These findings are also in line with evidence that revealed a lack of access to animal products and healthcare within communities [8] and a positive impact of community animal health programs on animal health outcomes [30,53]. 

### 4.1. Strengths and Limitations 

This pilot study was positively received, collected valuable information, and highlighted key areas for further investigation. The household survey was designed iteratively and adjusted according to feedback on the language and delivery of the survey in the local context. We used a household survey method, which is supported by the International Companion Animal Management Coalition as a method to assess public health risks related to animal populations and preventive health program impacts [54]. While this study was limited to One Health factors related to health risks associated with barriers to accessing animal healthcare and associated environmental health exposures (such as the risk of zoonotic disease), it may be applicable to other health risks that exist at the human–animal–environment interface within Aboriginal and Torres Strait Islander communities. 

We undertook an ecological study using household data, as this provided a good representation of One Health and allowed us to analyse data from multiple sectors together rather than analysing individual-level and separate sectoral data. However, in general, ecological studies have proven to be limited in their ability to draw conclusions [40]. Our sample included 7–10% of the households in each community with large amounts of missing data for some items, limiting the dataset and the approaches for further statistical analysis. Due to the iterative design of the data collection tool, the survey items on hunting and animal husbandry were only asked in two out of three communities and were not reported in the results. The limited questions regarding human and environmental health limited our ability to draw conclusions on the association between these exposures and animal health outcomes. These limitations are due to the nature of pilot studies, the limited time we had in each community, and the competing priorities on the ground (a simultaneous community-wide animal census and preventive health program). 

We used multiple data collection tools, which led to challenges in analysing the data due to the need to link two datasets to undertake a One Health analysis. By expanding the current dataset in future iterations, we could investigate further analysis to assess the associations between human, environmental, and animal health by means of fitting regression models or hypothesis testing. The development of additional indicators that are comparable and analysable across sectors would also be beneficial. Combining the data collection tools into one medium may be useful for future research projects and assist with the ease of delivery and subsequent analysis; however, to avoid duplicative data collection, this needs to be considered in the context of other data capture occurring; for example, the AMRRIC mobile app is used to capture animal population data for ongoing community animal health programs. 

### 4.2. Implications

This study highlights the need for more work to be conducted to bring the animal–human–environmental health sectors together to form a cohesive approach around data items, collection methods, and a framework for analysis to progress One Health beyond the siloed approach. It also highlights the need for ongoing and accessible community initiatives for animal healthcare and recognition of the importance of One Health for health and wellbeing. A one-size-fits-all approach is unlikely to be as effective as community-specific approaches that take into account community priorities, with local engagement and leadership needed to address this [29]. This community-driven approach is supported by the transdisciplinary nature of One Health, which recognises the need for multisectoral methods, community partnerships, and the recognition of traditional knowledge and practices [1,55]. Transdisciplinary approaches support community members, researchers, and policymakers, who use their varied knowledge and experiences to work together to address public health risks and break down the common siloed approach [56].

When considering community initiatives, the International Companion Animal Management Coalition and the Intergroup on the Welfare and Conservation of Animals support preventive population management rather than reactive control, taking a sustainable and long-term approach to animal health [57]. However, ongoing resourcing and training of local workforces are needed to improve service delivery, including education programs about animal ownership, health, and welfare [58]. Animal healthcare has been recommended as a cost-effective approach to controlling zoonoses in under-resourced communities; therefore, it should be supported by local, state, and federal governments that strive to control communicable diseases [59]. Effective animal population management can also assist with surveillance efforts to prevent and control emerging and exotic zoonotic diseases, particularly in the north of Australia [13]. Furthermore, integrated health systems are recommended to address public health risks, including emerging zoonotic diseases, with One Health providing an ideal framework for this [60]. However, community participation and leadership are essential to achieve sustainable change and improved health outcomes [61]. These initiatives can assist in addressing the Sustainable Development Goals; however, as the goals fall short of specifically addressing Indigenous groups, an assessment of the applicability of the indicators is warranted [31,62]. 

While this study had limitations related to data availability, the One Health data framework was useful in undertaking a One Health study with plans to further develop the framework and build on these findings. The inclusion of survey items regarding cultural and social determinants of health, the relationships between families and their animals, and further One Health factors would be beneficial to improving understanding and informing One Health approaches [15,63]. In line with the One Health principles that highlight the need for change in human behaviour, social science components regarding behaviour and experiences in relation to animal and environmental health practices would also be useful to inform One Health approaches [55]. The One Health data framework has the potential to be adapted to varying contexts and public health risks in other communities; however, consideration of the ownership and use of data is needed, particularly as it relates to Indigenous data sovereignty and governance principles [64,65].

## 5. Conclusions

This pilot study is the first study to investigate One Health within Aboriginal and Torres Strait Islander communities using a One Health data framework; the findings highlight that the concept of One Health in communities is still emerging. Communities identified a need for education programs around animal ownership and access to medicines and veterinary care. There is also a need to improve environmental health practices within households. To address this, sustainable resourcing and trained local workforces are needed, with policy support likely to assist. One Health approaches are likely to be effective in this setting, and leadership and input from the community are required to adopt a transdisciplinary approach and allow the incorporation of community priorities. These findings will be used to inform future research, including further development of the One Health data collection and analysis framework. 

## Figures and Tables

**Table 1:** Tests of association for healthy body condition score (BCS) (human and environmental health exposures vs. animal health outcome).

Healthy BCS				
	Unhealthy BCS N (%)	Healthy BCS N (%)	Total N (%)	Fisher’s Exact Test (*p*-Value)
**Human Health Exposures**				
>2 people per bedroom per household	1 (14.3)	5 (10.6)	6 (11.1)	1.00
≤2 people per bedroom per household	6 (85.7)	42 (89.4)	48 (88.9)	
Total	7 (100)	47 (100)	54 (100)	
Perceived crowding	1 (14.3)	12 (25.5)	13 (24.1)	1.00
No crowding	6 (85.7)	35 (74.5)	41 (75.9)	
Total	7 (100)	47 (100)	54 (100)	
Concerns for animal health	5 (71.4)	25 (52.1)	30 (54.5)	0.44
No concerns	2 (28.6)	23 (47.9)	25 (45.5)	
Total	7 (100)	48 (100)	55 (100)	
**Environmental Health Exposures**				
Household concerns	7 (100.0)	43 (87.8)	50 (89.3)	1.00
No concerns	0 (0.0)	6 (12.2)	6 (10.7)	
Total	7 (100)	49 (100)	56 (100)	
Animal crowding	1 (14.3)	15 (30.6)	16 (28.6)	0.66
No animal crowding	6 (85.7)	34 (69.4)	40 (71.4)	
Total	7 (100)	49 (100)	56 (100)	
Animal breeding	0 (0.0)	8 (16.3)	8 (14.3)	0.58
No breeding	7 (100.0)	41 (83.7)	48 (85.7)	
Total	7 (100)	49 (100)	56 (100)	

**Table 2:** Tests of association for healthy hair score (human and environmental health exposures vs. animal health outcome).

Healthy Hair Score				
	Unhealthy Hair Score N (%)	Healthy Hair Score N (%)	Total N (%)	Fisher’s Exact Test (*p*-Value)
**Human Health Exposures**				
>2 people per bedroom per household	3 (37.5)	3 (7.7)	6 (12.8)	0.05
≤2 people per bedroom per household	5 (62.5)	36 (92.3)	41 (87.2)	
Total	8 (100)	39 (100)	47 (100)	
Perceived crowding	2 (25.0)	9 (23.1)	11 (23.4)	1.00
No crowding	6 (75.0)	30 (76.9)	36 (76.6)	
Total	8 (100)	39 (100)	47 (100)	
Concerns for animal health	5 (62.5)	22 (55.0)	27 (56.2)	1.00
No concerns	3 (37.5)	18 (45.0)	21 (43.8)	
Total	8 (100)	40 (100)	48 (100)	
**Environmental Health Exposures**				
Household concerns	7 (87.5)	36 (87.8)	43 (87.8)	1.00
No concerns	1 (12.5)	5 (12.2)	6 (12.2)	
Total	8 (100)	41 (100)	49 (100)	
Animal crowding	2 (25.0)	13 (31.7)	15 (30.6)	1.00
No animal crowding	6 (75.0)	28 (68.3)	34 (69.4)	
Total	8 (100)	41 (100)	49 (100)	
Animal breeding	1 (12.5)	7 (17.1)	8 (16.3)	1.00
No breeding	7 (87.5)	34 (82.9)	41 (83.7)	
Total	8 (100)	41 (100)	49 (100)	

**Table 3:** Tests of association for healthy tick and flea score (human and environmental health exposures vs. animal health outcome).

Healthy Tick and Flea Scores				
	Unhealthy Tick and Flea Score N (%)	Healthy Tick and Flea Score N (%)	Total N (%)	Fisher’s Exact Test (*p*-Value)
**Human Health Exposures**				
>2 people per bedroom per household	2 (16.7)	3 (9.4)	5 (11.4)	0.60
≤2 people per bedroom per household	10 (83.3)	29 (90.6)	39 (88.6)	
Total	12 (100)	32 (100)	44 (100)	
Perceived crowding	2 (16.7)	8 (25.0)	10 (22.7)	0.70
No crowding	10 (83.3)	24 (75.0)	34 (77.3)	
Total	12 (100)	32 (100)	44 (100)	
Concerns for animal health	9 (75.0)	15 (45.5)	24 (53.3)	0.10
Not concerns	3 (25.0)	18 (54.5)	21 (46.7)	
Total	12 (100)	33 (100)	45 (100)	
**Environmental Health Exposures**				
Household concerns	12 (92.3)	28 (84.9)	40 (87.0)	0.66
No concerns	1 (7.7)	5 (15.1)	6 (13.0)	
Total	13 (100)	33 (100)	46 (100)	
Animal crowding	2 (15.4)	12 (36.4)	14 (30.4)	0.29
No animal crowding	11 (84.6)	21 (63.6)	32 (69.6)	
Total	13 (100)	33 (100)	46 (100)	
Animal breeding	2 (15.4)	6 (18.2)	8 (17.4)	1.00
No breeding	11 (84.6)	27 (81.8)	38 (82.6)	
Total	13 (100)	33 (100)	46 (100)	

## Data Availability

The dataset is subject to ethical considerations and is thus not available for public use.

## Supplementary Materials

The following supporting information can be downloaded at: https://www.mdpi.com/article/10.3390/ijerph20146416/s1, Table S1: Data collection items; Table S2: Exposure and outcome variables; Table S3: Fisher’s exact test; Table S4: One Health at the household level.
