# Supplementary material for: One Health and Australian Aboriginal and Torres Strait Islander Communities: A One Health Pilot Study
Source: Int J Environ Res Public Health. 2023 Jul 20;20(14):6416. doi: 10.3390/ijerph20146416 (PMC10378938; doi:10.3390/ijerph20146416)
Supplement: Supplementary file 1 [file ijerph-20-06416-s001.zip › ijerph-2432979-supplementary.pdf]

Supplementary Material

Supplementary Table S1: Data collection items

Identifying questions			
House address and community?<br>Free response<br>Do Aboriginal and/or Torres Strait Islander people live in this household?<br>Yes, No, Undisclosed			
**Human health**	**Animal health**	**Environmental health**	**One Health concept**
How many people live in the household?<br>1-10+ people<br><br>How many bedrooms does the household have?<br>1-5+ bedrooms<br>Don't know<br><br>Is the household crowded?<br>Yes a lot<br>Yes a fair bit<br>Yes a little bit<br>No<br>Don't know	Animal health assessments:<br>Species (dog or cat)<br>Breed<br>Size<br>Sex<br>Age group<br>Desexing status<br>Body condition score<br>Hair score<br>Tick and flea score<br>Other problems<br>Treatments delivered	Do you have any concerns with your household environment? If yes, what?<br>Water supply/quality<br>Containing animals (fencing)<br>Yard maintenance (grass)<br>Animal mess (carcasses/faeces)<br>Waste management (rubbish)<br>Washing and hygiene facilities<br>Pests (rodents, insects, parasites)<br>Wild animals (horses)<br>Power supply/electricity<br>Other (free response)	Do animals affect the health of people living in your household?<br>Yes a lot<br>Yes a fair bit<br>Yes a little bit<br>No<br>Don't know<br><br>If yes, how?<br>Itchy animals make people itchy<br>Sick animals make people sick<br>Injury from animals (bites, scratches<br>Affects wellbeing (sad when animals are sick or injured)<br>Don't know<br>Other (free response)
Are you worried about the health of your animals?<br>Yes a lot<br>Yes a fair bit<br>Yes a little bit<br>No<br>Don't know<br>If yes, what are your concerns?<br>Disease<br>Injury<br>Too many animals<br>Can't access animal health care<br>Don't know<br>Other (free response)	Does the community need animal health care? If yes, what?<br>Vet based in community<br>Vet visiting the community<br>Animal medicines available<br>Community animal health workers<br>Regular and ongoing program<br>Don't need animal health care<br>Controlling wild animals<br>Don't know<br>Other (free response)		Does the environment affect the health of people living in your household?<br>Yes a lot<br>Yes a fair bit<br>Yes a little bit<br>No<br>Don't know<br><br>If yes, how?<br>Impacts of wild animals (horses, pigs etc.)<br>Affects health of people<br>Affects health of dogs and cats<br>Weather events (storms, fires, floods)<br>Impact of climate change<br>Don't know<br>Other (free response)

Supplementary Table S2: Exposure and outcome variables

**Human health (exposures)**		**Environmental health (exposures)**		**Animal health (outcomes)**	
Variable	*Options*	*Variable*	*Options*	*Variable*	*Options*
Household crowding	>2 people per bedroom per household (crowded)<br>≤2 people per bedroom per household (not crowded)	Household environmental concerns	Concerns with household environment (at least one concern)<br>No concerns (no)	Body condition score (BCS)	Unhealthy BCS (thin BCS 1-3 and overweight BCS 7-9)<br>Healthy BCS (BCS 4-6)
Perceived crowding	Crowded (yes to feels crowded)<br>Not crowded (no)	Animal crowding	Animal crowding (>4 animals per household)<br>No animal crowding (1-4 animals per household)	Hair score	Unhealthy hair score (hair score 2-6)<br>Healthy hair score (hair score 1)
Concerns for animal health	Concerns for animal health (yes to has concerns)<br>No concerns (no)	Animal breeding	Breeding (puppies and/or kittens present)<br>No breeding (no litters)	Tick and flea score	Unhealthy tick and flea score (tick and flea score 2-4)<br>Healthy tick and flea score (tick and flea score 1)

Supplementary Table S3: Fisher's exact test

**Healthy BCS**				
	Unhealthy BCS O (E)*	Healthy BCS O (E)*	Total	Fisher's exact test (p-value)
Human health exposures				
>2 people per bedroom per household	1 (0.8)	5 (5.2)	6	1.00
≤2 people per bedroom per household	6 (6.2)	42 (41.8)	48	
Total	7	47	54	
Perceived crowding	1 (1.7)	12 (11.3)	13	1.00
No crowding	6 (5.3)	35 (35.7)	41	
Total	7	47	54	
Concerns for animal health	5 (3.8)	25 (26.2)	30	0.44
No concerns	2 (3.2)	23 (21.8)	25	
Total	7	48	55	
Environmental health exposures				
Household concerns	7 (6.3)	43 (43.8)	50	1.00
No concerns	0 (0.8)	6 (5.3)	6	
Total	7	49	56	
Animal crowding	1 (2.0)	15 (14.0)	16	0.66
No animal crowding	6 (5.0)	34 (35.0)	40	
Total	7	49	56	
Animal breeding	0 (1.0)	8 (7.0)	8	0.58
No breeding	7 (6.0)	41 (42.0)	48	
Total	7	49	56	
**Healthy hair score**				
	Unhealthy hair score O (E)*	Healthy hair score O (E)*	Total	Fisher's exact test (p-value)
Human health exposures				
>2 people per bedroom per household	3 (1.0)	3 (5.0)	6	0.05
≤2 people per bedroom per household	5 (7.0)	36 (34.0)	41	
Total	8	39	47	
Perceived crowding	2 (1.9)	9 (9.1)	11	1.00
No crowding	6 (6.1)	30 (29.9)	36	
Total	8	39	47	
Concerns for animal health	5 (4.5)	22 (22.5)	27	1.00
No concerns	3 (3.5)	18 (17.5)	21	

Total	8	40	48	
Environmental health exposures				
Household concerns	7 (7.0)	36 (36.0)	43	1.00
No concerns	1 (1.0)	5 (5.0)	6	
Total	8	41	49	
Animal crowding	2 (2.4)	13 (12.6)	15	1.00
No animal crowding	6 (5.6)	28 (28.4)	34	
Total	8	41	49	
Animal breeding	1 (1.3)	7 (6.7)	8	1.00
No breeding	7 (6.7)	34 (34.3)	41	
Total	8	41	49	
**Healthy tick and flea scores**				
	Unhealthy tick and flea score O (E)*	Healthy tick and flea score O (E)*	Total	Fisher's exact test (p-value)
Human health exposures				
>2 people per bedroom per household	2 (1.4)	3 (3.6)	5	0.60
≤2 people per bedroom per household	10 (10.6)	29 (28.4)	39	
Total	12	32	44	
Perceived crowding	2 (2.7)	8 (7.3)	10	0.70
No crowding	10 (9.3)	24 (24.7)	34	
Total	12	32	44	
Concerns for animal health	9 (6.4)	15 (17.6)	24	0.10
No concerns	3 (5.6)	18 (15.4)	21	
Total	12	33	45	
Environmental health exposures				
Household concerns	12 (11.3)	28 (28.7)	40	0.66
No concerns	1 (1.7)	5 (4.3)	6	
Total	13	33	46	
Animal crowding	2 (4.0)	12 (10.0)	14	0.29
No animal crowding	11 (9.0)	21 (23.0)	32	
Total	13	33	46	
Animal breeding	2 (2.3)	6 (5.7)	8	1.00
No breeding	11 (10.7)	27 (27.3)	38	
Total	13	33	46	

* O: Observed frequency; E: Expected frequency

Supplementary Table S4: One Health at household level

Questions	Number (N)	Percentage (%)
**Do Aboriginal and/or Torres Strait Islander people live in this household?**		
Yes	81	98.8
No	1	1.2
Missing	0	0.0
Total	82	100.0
One Health concept		
**Do animals affect the health of people living in your household?**		
No	38	46.3
Yes	34	41.5
Don't know	1	1.2
Missing	9	11.0
Total	82	100.0
**If yes, how? (can choose multiple options)**		
Itchy animals make people itchy	9	11.0
Sick animals make people sick	12	14.6
Injury from animals (bites, scratches	4	4.9
Affects wellbeing (sad when animals are sick or injured)	13	15.8
Other (comments)	26	31.7
**Does the environment affect the health of people living in your household?**		
No	24	29.3
Yes	19	23.2
Don't know	3	3.7
Missing	36	43.9
Total	82	100.0
**If yes, how? (can choose multiple options)**		
Impacts of wild animals (horses, pigs etc.)	3	3.7
Affects health of people	6	7.3
Affects health of dogs and cats	1	1.2
Impact of climate change	3	3.7
Don't know	2	2.4
Other (comments)	17	20.7
Human Health		
**How many people live in the household?**		
1-2	16	19.5
3-4	26	31.7
5-6	17	20.7
7-8	13	15.8
9+	9	11.0
Missing	1	1.2
Total	82	100.0
**How many bedrooms does the household have?**		
1	1	1.2
2	13	15.8
3	22	26.8
4	32	39.0
5+	10	12.2
Don't know	1	1.2

Missing	3	3.7
Total	82	100.0
**Crowding**		
Not crowded (≤2 people per bedroom per household)	68	82.9
Crowded (>2 people per bedroom per household)	11	13.4
Missing	3	3.7
Total	82	100.0
**Is the household crowded?**		
No	61	74.4
Yes	17	20.7
Don't know	1	1.2
Missing	3	3.7
Total	82	100.0
**Are you worried about the health of your animals?**		
No	37	45.1
Yes	43	52.4
Missing	2	2.4
Total	82	100.0
**If yes, what are your concerns? (can choose multiple options)**		
Disease	25	30.5
Injury	9	11.0
Too many animals	5	6.1
Other (comments)	38	46.3
Environmental health		
**Do you have any concerns with your household environment?**		
Yes	71	86.6
No	11	13.4
Total	82	100.0
**If yes, what? (can choose multiple options)**		
Water and power supply/quality	7	8.5
Containing animals (fencing)	47	57.3
Yard and rubbish management	4	4.9
Animal mess (carcasses/faeces)	11	13.4
Washing and hygiene facilities	1	1.2
Pests (rodents, insects, parasites)	52	63.4
Wild animals (horses)	24	29.3
Other (comments)	61	74.4
**Household owns cats and dogs**		
Yes	67	81.7
No	15	18.3
Total	82	100.0
**Cats and dogs per house**		
0	15	18.3
1-4	48	58.5
5-8	14	17.1
9-12	5	6.1
Total	82	100.0
**Litters present**		
Puppies or kittens present	8	11.9

No litters	59	88.1
Total	67*	100.0
Animal health		
**Body condition score household average**		
Thin (BCS 1-3)	3	4.5
Ideal (BCS4-6)	49	73.1
Overweight (BCS 7-9)	4	6.0
Missing	11	16.4
Total*	67*	100.0
**Hair score household average**		
No hair loss (full coat)	41	61.2
Up to 20% hair loss	8	11.9
Missing	18	26.9
Total*	67*	100.0
**Tick score household average**		
No ticks	35	52.2
Mild ticks	9	13.4
Moderate ticks	1	1.5
Missing	22	32.8
Total*	67*	99.9
**Flea score household average**		
No fleas	36	53.7
Mild fleas	7	10.5
Missing	24	35.8
Total*	67*	100.0
**Does the community need animal health care? If yes, what? (can choose multiple options)**		
Vet based in community	12	14.6
Vet visiting the community	33	40.2
Animal medicines available	42	51.2
Community animal health workers	5	6.1
Regular and ongoing program	26	31.7
Don't need animal health care	2	2.4
Controlling wild animals	3	3.7
Don't know	2	2.4
Other (comments)	66	80.5

* Households with no animals excluded
